# A novel TRPC6 variant (c.131C>T, p.(Pro44Leu)) associated with focal segmental glomerulosclerosis: a case report

**DOI:** 10.3389/fgene.2026.1913168

**Published:** 2026-08-10

**Authors:** Fan Yang, Xiaoqi Wang, Yan Li, Kaijie Su, Ran Ding, Linlin Wu, Guorong Ma, Jianjie Yang, Zhongxin Li

**Affiliations:** 1 Department of Nephrology, Beijing Luhe Hospital Affiliated to Capital Medical University, Beijing, China; 2 Department of Pathology, Beijing Luhe Hospital Affiliated to Capital Medical University, Beijing, China

**Keywords:** case report, focal segmental glomerulosclerosis, novel variant, podocytopathy, TRPC6

## Abstract

**Background:**

Pathogenic variants in the transient receptor potential cation channel subfamily C member 6 (TRPC6) cause autosomal dominant focal segmental glomerulosclerosis (FSGS). We report a patient with early-onset FSGS carrying a novel *TRPC6* variant not previously described.

**Case presentation:**

A 21-year-old Chinese male presented with proteinuria (3.17g/24h) and mild renal insufficiency (Cr 103 μmol/L). Renal biopsy confirmed FSGS, not otherwise specified (NOS). Genetic testing was initially declined due to cost concerns. He received losartan, dapagliflozin, strict salt restriction, and ambrisentan, achieving proteinuria reduction to 0.7g/24h without immunosuppression. Two years later, genetic testing identified a novel *TRPC6* variant: c.131C>T p.(Pro44Leu), extremely rare in public databases and classified as a variant of uncertain significance.

**Conclusion:**

This is the first report of the *TRPC6* p.Pro44Leu variant, expanding the variant spectrum of *TRPC6*-associated FSGS. The clinical decision to withhold immunosuppression was guided primarily by the patient’s phenotype (young age, sub-nephrotic proteinuria, FSGS-NOS, and no secondary causes); the *TRPC6* variant, although classified as a VUS, provided supportive evidence for a genetic etiology and reinforced this management approach. This case demonstrates that genetic testing can guide personalized management in young patients with FSGS, offering a practical framework for avoiding unnecessary treatment-related morbidity. This case also illustrates that cost and psychological barriers can delay genetic diagnosis and highlights the value of early supportive therapy in genetic FSGS.

## Introduction

A 21-year-old Chinese male presented with proteinuria (3.17 g/24h) and mild renal insufficiency (creatinine 103 μmol/L). Renal biopsy confirmed focal segmental glomerulosclerosis (FSGS), not otherwise specified. Genetic testing identified a novel *TRPC6* variant, c.131C>T p.(Pro44Leu). The patient achieved significant proteinuria reduction with supportive therapy alone.

Focal segmental glomerulosclerosis (FSGS) is a common cause of steroid-resistant nephrotic syndrome, often progressing to end-stage kidney disease (Rosenberg and Kopp, 2017; D'Agati et al., 2011). Genetic forms account for many early-onset or familial cases. The international consensus indicates FSGS is a pattern of glomerular injury from diverse etiologies converging on podocyte dysfunction (Romagnani et al., 2025). Some patients present with sub-nephrotic proteinuria and respond poorly to immunosuppression, making etiologic identification essential (Vriese et al., 2018).

*TRPC6* encodes a non-selective cation channel localized at the podocyte slit diaphragm (Reiser et al., 2005). Since 2005, more than 40 pathogenic *TRPC6* variants have been reported, mostly gain-of-function missense variants (Reiser et al., 2005; Winn et al., 2005; Mukerji et al., 2007; Miao and Zand, 2025; Wooden et al., 2025; Liu et al., 2021). We report a patient with early-onset FSGS carrying a novel *TRPC6* variant, c.131C>T p.(Pro44Leu), not previously described in the literature.

### Case presentation

A 21-year-old Chinese male (self-identified) presented with throat discomfort and frothy urine. He had no history of hypertension, diabetes, or kidney disease in his family. He denied smoking or alcohol use. Physical examination showed blood pressure 119/77 mmHg, body mass index 20.58 kg/m^2^, and no edema. Laboratory investigations revealed 24-h urinary protein 3.17 g (reference range <0.15 g/24h), serum albumin 47.4 g/L (reference range 40–55 g/L), creatinine 103 μmol/L (reference range 57–97 μmol/L), and cholesterol 8.75 mmol/L (reference range 3.11–5.17 mmol/L). Secondary etiology screening was unremarkable, including negative results for viral serologies, serum protein electrophoresis, antinuclear antibodies, and complement levels. The patient had no history of medication use associated with FSGS (e.g., pamidronate, interferon, lithium), and no history of low birth weight or prematurity.

**FIGURE 1:**
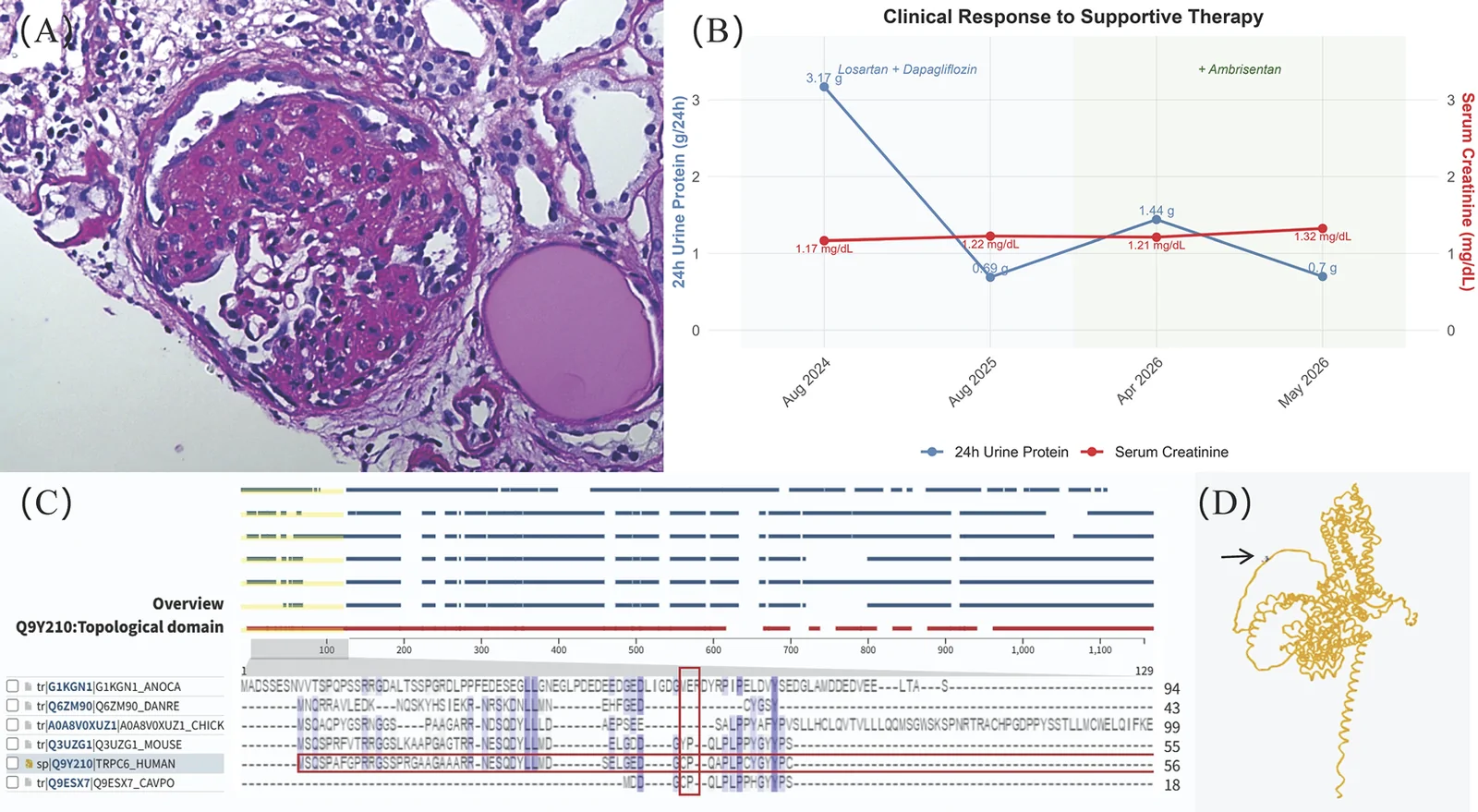
**(A)** Light microscopy (Periodic acid-Schiff stain, high magnification, ×400) shows a glomerulus with segmental glomerulosclerosis. Surrounding atrophic tubules and intratubular protein casts are also visible. **(B)** Clinical timeline of the patient from initial presentation to genetic diagnosis. **(C)** Multiple sequence alignment of *TRPC6* orthologs. Pro44 (red box) is conserved across mammals (human, mouse, guinea pig) but absent or non-conserved in non-mammalian species (lizard, chicken, zebrafish), indicating mammal-specific conservation. **(D)** Three-dimensional structure of TRPC6 (AlphaFold AF-Q9Y210-F1). Pro44 (arrow) is located on the protein surface (RSA = 72.9%) within a loop region, suggesting involvement in protein-protein interactions rather than structural core maintenance.

Renal biopsy was performed. Immunofluorescence demonstrated IgA (+), IgM (+), and C3 (+∼++). Light microscopy revealed FSGS, not otherwise specified (15 glomeruli; one with ischemic wrinkling, one with segmental sclerosis; approximately 5% tubular atrophy and interstitial fibrosis) (Figure 1A).

Although genetic testing was initially recommended as a means to establish an etiologic diagnosis and guide therapeutic decision-making, the patient declined due to cost concerns. Two years later, following repeated counseling on the potential clinical utility of genetic results, he provided consent. Exome sequencing (KingMed Diagnostics) identified a heterozygous missense *TRPC6* variant: NM_004621.6:c.131C>T p.(Pro44Leu) in exon 1 (chr11:101,454,104 G>A, GRCh37). Sanger sequencing confirmed the variant.

The variant is extremely rare in public databases. It is present at very low frequency in gnomAD (allele frequency: 4.0 × 10^−6^; 1/234,090 alleles in gnomAD exomes), and at slightly higher frequencies in other well-controlled databases (e.g., up to 7.7 × 10^−5^ in AllofUs). It has not been reported in ClinVar, LOVD, or dbSNP. To our knowledge, no previous literature has described this variant in association with FSGS. An adjacent variant (p.Pro44Ser) is present at very low population frequency in public databases (gnomAD cumulative allele frequency: 0.000138, with only one homozygote reported among over 1.6 million alleles). Using the Franklin platform (2022 ACMG guidelines), the variant met PM2 (extremely low population frequency) and BP4 (most *in silico* tools predict benign; aggregate score 0.149), whereas SIFT and MutationTaster predicted damaging. It was classified as a variant of uncertain significance (Supplementary Table 1).

Multiple sequence alignment revealed Pro44 is conserved across mammals but absent in non-mammalian vertebrates, suggesting mammal-specific acquisition (Figure 1C). Structural analysis (AlphaFold AF-Q9Y210-F1) showed Pro44 located on the protein surface (relative solvent accessibility 72.9%), suggesting involvement in protein-protein interactions (Figure 1D).

No immunosuppressants were used. The patient received losartan, dapagliflozin, ambrisentan, and strict dietary sodium restriction. At follow-up, 24-h urinary protein decreased to 0.7 g with stable renal function. The clinical timeline is summarized in Figure 1B.

## Discussion

We report a Chinese individual with biopsy-proven FSGS carrying a novel *TRPC6* variant, c.131C>T p.(Pro44Leu), which is extremely rare in public databases (gnomAD allele frequency: 4.0 × 10^−6^). The predominance of benign predictions from most *in silico* tools, together with the lack of functional studies, supports its classification as a variant of uncertain significance. The patient achieved significant proteinuria reduction with supportive therapy alone, suggesting a mild disease trajectory. A recent large-scale study by Wooden et al. (Wooden et al., 2025) analyzing 64 patients from 39 families with *TRPC6*-associated podocytopathy identified 21 unique missense variants and defined three distinct protein regional hotspots, further supporting the clinical significance of variants in this gene and providing a valuable reference for interpreting novel variants such as p.Pro44Leu. Additionally, a Chinese pedigree study identified a gain-of-function *TRPC6* Q134P variant in a late-onset FSGS family, demonstrating that functional alterations in TRPC6 can produce a mild, delayed phenotype (Liu et al., 2021). More recently, a Chinese cohort study of adult FSGS patients reported that genetic mutations were associated with better short-term kidney prognosis but no differences in long-term prognosis (Min et al., 2025). These observations highlight the importance of functional validation and population-specific reference data for interpreting *TRPC6* variants across diverse ethnic groups. We acknowledge that a VUS should not guide clinical decisions alone. Here, the decision to avoid immunosuppression was phenotype-driven; the genetic finding was retrospective support. This practice is consistent with published guidance that a VUS should not be used alone for clinical decisions, while recognizing that genetic testing can still inform management when integrated with phenotype (Savige, 2024).

Based on the podocytopathy consensus (Romagnani et al., 2025), this patient’s phenotype (body mass index 20.58, no diabetes, no low birth weight) makes adaptive factors unlikely; autoimmune disease typically presents with nephrotic syndrome. Thus, genetic podocytopathy is consistent.

A practical diagnostic approach should consider genetic FSGS in patients presenting with onset under 30 years, family history of kidney disease, biopsy findings showing FSGS not otherwise specified, sub-nephrotic proteinuria, or lack of response to immunosuppressive therapy (Romagnani et al., 2025; Vriese et al., 2018). In such patients, early genetic testing can help distinguish monogenic forms from primary FSGS, thereby avoiding unnecessary courses of corticosteroids or calcineurin inhibitors, which carry substantial side effects. Even in resource-limited settings where genetic testing is not immediately accessible, the clinical phenotype alone may justify a trial of supportive therapy before initiating immunosuppression. Our patient exemplifies this approach: supportive care was initiated upfront; genetic testing later provided supportive evidence and reinforced this strategy. RAS inhibition combined with dapagliflozin improves hyperfiltration in remnant nephrons through both hemodynamic and non-hemodynamic mechanisms, with robust evidence supporting their use in CKD, including patients with FSGS lesions (Wheeler et al., 2022). Endothelin receptor antagonists have demonstrated antiproteinuric effects in chronic kidney disease. In IgA nephropathy and Alport syndrome, a hereditary glomerular disease that can present with FSGS-like lesions, selective endothelin A receptor antagonists including atrasentan and ambrisentan have shown significant proteinuria reduction with preserved kidney function (Song et al., 2024; Shi et al., 2025). Mechanistically, endothelin A receptor blockade attenuates glomerular endothelial injury, reduces mesangial proliferation, and modulates profibrotic pathways such as AKT/GSK-3β signaling (Bhadange and Gaikwad, 2026). Although sparsentan, a dual endothelin-angiotensin receptor antagonist, showed superior proteinuria reduction in FSGS compared to irbesartan (Rheault et al., 2023), it remains unavailable in China. Given the established renoprotective effects of RAS inhibition, SGLT-2 inhibition, and endothelin receptor antagonists in proteinuric glomerular diseases, including genetic kidney diseases that exhibit FSGS lesions, we applied this evidence to our patient by adding ambrisentan to the supportive regimen.

The p.Pro44Leu variant lies upstream of the first ankyrin repeat domain (residues 97–316), outside the classical FSGS mutation hotspot within the ankyrin repeats themselves. Its surface-exposed location—predicted to face outward from the channel core in the homotetrameric TRPC6 configuration—suggests involvement in protein-protein interactions rather than pore function. Together with the observed interaction network between TRPC6 and slit diaphragm proteins (NPHS2 and NPHS1) and literature reports of nephrin binding to the TRPC6 N-terminus (Kanda et al., 2011), we hypothesize that p.Pro44Leu alters local surface conformation, interfering with slit diaphragm protein interactions. Additionally, RNF24 regulates TRPC6 trafficking via the ankyrin repeat domain (Lussier et al., 2008); although Pro44 is upstream, it is plausible that this variant may affect ankyrin repeat domain conformation, disrupting RNF24-mediated regulation. However, this mechanistic interpretation remains hypothetical, and direct experimental evidence is needed to confirm pathogenicity.

The identification of a *TRPC6* variant in this patient has immediate clinical implications and opens future therapeutic opportunities. An oral *TRPC6* inhibitor (BI 764198) recently demonstrated a 35% proteinuria response rate in a phase 2 trial (Trachtman et al., 2026). For this patient and others with *TRPC6*-associated FSGS, genetic diagnosis not only enables avoidance of unnecessary immunosuppression but also may inform eligibility for emerging targeted therapies, should they become available. In regions where these agents are not yet accessible, genetic testing still offers value by informing prognosis, guiding family counseling, and supporting informed shared decision-making between clinicians and patients.

This report expands the variant spectrum of *TRPC6*-associated FSGS and provides a practical example of how genetic testing can directly inform clinical management in young patients with unexplained FSGS. The integration of genetic diagnosis into routine nephrology practice informs personalized therapeutic decision-making, minimizes unnecessary treatment-related harm, and facilitates patient eligibility for emerging targeted therapies. We advocate for early genetic testing in suspected monogenic FSGS to optimize patient outcomes and resource utilization.

### Clinical takeaway

This case illustrates that structural modeling of novel variants can provide supportive insights that may inform clinical reasoning, even in the absence of functional studies. The surface-exposed, non-core location of p.Pro44Leu was consistent with the patient’s mild clinical phenotype. We suggest that structural prediction should be considered alongside clinical features in the interpretation of genetic variants in FSGS to guide risk stratification and therapeutic decision-making.

## Limitations

This study has several limitations. First, functional studies (e.g., patch-clamp, calcium imaging) were not performed to validate the functional impact of the variant. Second, the patient declined family screening, so the origin of the variant could not be determined. Third, available cryo-EM structures lack the N-terminal region containing Pro44; therefore, the AlphaFold-predicted structure awaits experimental validation. Fourth, the follow-up period is relatively short (21 months), and long-term prognosis requires further observation. Despite these limitations, the extreme rarity, evolutionary conservation, surface-exposed location, and phenotypic consistency suggest that this variant could be a candidate susceptibility variant for FSGS. However, the clinical phenotypic association suggested by this case should not be equated with definitive molecular pathogenic validation, which would require functional and familial segregation studies.

## Patient perspective

The patient initially declined genetic testing due to high cost and uncertainty. After repeated counseling over 2 years, he consented. He reported good adherence to supportive therapy and salt restriction, as proteinuria reduction reinforced his confidence. He declined family segregation analysis, concerned that his parents might feel guilty or anxious, highlighting psychological challenges in genetic diagnosis.

## Data Availability

The datasets presented in this article are not readily available because of ethical and privacy restrictions. Requests to access the datasets should be directed to the corresponding author/s. The TRPC6 variant (NM_004621.6:c.131C>T) is accessible in ClinVar under accession number SCV007596794.
